# Risks associated with communication delays in infants from underserved South African communities

**DOI:** 10.4102/phcfm.v7i1.841

**Published:** 2015-09-08

**Authors:** Jeannie van der Linde, De Wet Swanepoel, Frances P. Glascoe, E.M. Louw, Jannie F.M. Hugo, Bart Vinck

**Affiliations:** 1 Department of Speech-Language Pathology and Audiology, University of Pretoria, South Africa; 2 Ear Sciences Centre, School of Surgery, University of Western Australia, Nedlands, Australia; 3 Ear Science Institute Australia, Subiaco, Australia; 4 Department of Pediatrics, Vanderbilt University, United States; 5 Department of Statistics, University of Pretoria, South Africa; 6 Department of Family Medicine, University of Pretoria, South Africa; 7 Department of Speech-Language Pathology and Audiology, Ghent University, Belgium

## Abstract

**Background:**

For optimal development young children need warm, responsive, enriched and communicative environments for learning social, language, and other skills. Infants and toddlers exposed to psychosocial risk lack enriched environments and may present with communication delays.

**Aim:**

To investigate the relationship between psychosocial risks and communication delays in infants from underserved communities in South Africa.

**Setting:**

Primary healthcare facilities in Tshwane district, South Africa.

**Methods:**

A parent interview and Rossetti Infant Toddler Language Scales were used to collect data from caregivers of 201 infants aged 6–12 months, selected through convenience sampling. Associations between communication delays and risks were determined (Chi-square and Fisher's exact tests). A log-linear model analysis was used to model the simultaneous effect of significant risks on the probability of having communication delays.

**Results:**

Communication delays were present in 13% of infants. Infants with two or more siblings, born from mothers aged 18–29 years who own their house, had a 39% chance of presenting with communication delays.

**Conclusion:**

Developmental screening and early intervention is important in primary healthcare contexts in South Africa, as a clear relationship has been established between three risk factors and communication delays in infants.

**Contexte:**

**Risques associés à des retards de communication verbale chez les nourrissons des communautés sud-africaines non desservies.**

Pour s'épanouir complètement les jeunes enfants ont besoin d'un environnement chaud, réceptif, enrichi et communicatif pour apprendre le langage social et d'autres compétences. Les nourrissons et les tout-petits exposés à des risques psychosociaux souffrent d'un manque d'environnements enrichissants et pourraient souffrir de retards de communication verbale.

**Objectif:**

Pour étudier la relation entre les risques psychosociaux et les retards de communication verbale chez les nourrissons des communautés non desservies en Afrique du Sud.

**Lieu:**

Services de soins primaires dans le district de Tshwane, en Afrique du Sud.

**Méthodes:**

Une entrevue avec les parents et l'Echelle de Compétence linguistique pour les Nourrissons de Rossetti ont été utilisés pour rassembler les données de 201 nourrissons de 6 à 12 mois provenant de leurs gardiens, sélectionnés au moyen d'échantillonnages de proximité. On a remarqué un lien entre les retards et les risques de communication verbale (tests Chi carré et de Fisher). On a utilisé un modèle d'analyse log-linéaire pour modéliser l'effet simultané des risques importants sur la probabilité d'avoir des retards de communication verbale.

**Résultats:**

On a trouvé des retards de communication verbale chez 13% des nourrissons. Les nourrissons qui vivent avec deux frères ou sœurs ou plus, nés de mères âgées de 18 à 29 ans qui ont leur propre maison, avaient 39% plus de chance d'avoir des retards de communication verbale.

## Introduction

For optimal development young children need a warm, responsive, enriched and communicative environment for learning social, language and other skills.^1^ Infants and young children exposed to risk conditions may present with developmental delays or disorders that may ultimately impact socio-emotional, educational and vocational outcomes.^2^ These risks include any potential factors that affect a child's ability to interact with his or her environment,^3,4^ which in turn results in developmental delays or disorders.

Communication delays are most prevalent in children under the age of three years.^4^ If communication delays remain undetected, this negatively impacts later educational and social performance, has long-term financial implications, and results in future delays or disorders.^5,6^ As a result of environmental factors such as unemployment, limited medical resources, lack of educational services, violence, crime and HIV infection and AIDS,^2^ the prevalence of communication delays or disorders is increasing.

In the United Kingdom an incidence of speech and language disability of 5.6% has been reported in children aged 0–2 years.^7^ Similar findings reported in a systematic review which included several countries indicated a median prevalence of 5% for speech and language delays in children of two years of age.^8^ In developing countries such as South Africa the prevalence of communication delays will probably be higher, due to more biological and psychosocial risks such as poverty, violence, nutritional deficiencies, HIV infection and substance abuse.^9^ Advanced or very young maternal age,^10^ lack of parent-child interaction,^11^ low parental educational levels, poor parental mental and physical health, insufficient parental coping strategies and confidence^12^ are pervasive risk factors characteristic of South Africa.^9^ These risks are likely to predispose infants to developmental delay.

Limited parental education negatively impacts communication acquisition in infants and young children due to a lack of parental knowledge and stimulation during the infants’ early years.^13^ Approximately 16% of adults (20 years or older) in South Africa are functionally illiterate, 34% have completed secondary levels of education only and 29% have completed Grade 12.^14^ Almost half of South Africans are deemed poor (45.5%), with 20% living in extreme poverty.^15^ Living in poor conditions restricts the quality and quantity of prenatal care, placing the unborn infant at risk of low birthweight and prematurity.^16^ Risk factors such as poverty and low parental education can occur in isolation or in combination, and it is generally accepted that an increase in the number of risk factors results in an increase in developmental risk to the infant.^3^

The impact of risk factors such as parental education at a level less than high school, limited social support, poverty and more than three children in the home is difficult to establish in infants before the ages of 2 to 3 years.^17^ Still, the most important phase of communication acquisition and development occurs between 8 and 24 months.^5,18,19^ Wide-ranging prevalence of language delay with high rates of spontaneous resolution have been reported.^5,8,20^ This illustrates the variability in the emergence of language skills, which in turn complicates the evaluation of infants’ and young children's communication development.^5^

The first two years of life are crucial in communication acquisition and development, and since the emergence of communication skills reportedly varies between individuals,^5^^,^^8^^,^^20^ the link between early risk factors and communication delays may provide reliable indicators to improve early detection. This might be especially informative in underserved or disadvantaged communities in countries like South Africa, where infants are exposed to multiple psychosocial risks along with health risks such as poverty, limited healthcare services and HIV infection and AIDS.^9^

In a previous study conducted across a spectrum of disadvantaged and advantaged urban communities in Melbourne, Australia, early risk factors could only explain 7% of the variation in language skills at the age of two years.^20^ A few South African studies have reported on risks and communication delays, but only in specific target populations such as infants with cleft lip and palate and babies with dysphagia.^21,22^ However, the relationships between risk factors and communication delays still need to be explored for infants in South Africa. More specifically, the relationships between risks and communication delays should be explored in underserved communities, which are deemed the poorest, most disadvantaged in the country, lack adequate public healthcare services and represent almost 50% of the population.^15,23^

Preventative strategies such as developmental screening or surveillance and intervention can be implemented from birth onwards to compensate for the risks, to eliminate or reduce the resultant communication delays. Early intervention in underserved communities is, however, hindered by financial constraints and a lack of resources to implement family-centred services.^24^ Identifying risk factors that may predispose to communication delays or disorders in infants in underserved communities is an important priority to strengthen primary prevention strategies. The objective of the current study was therefore to investigate the relationship of certain environmental risks in an underserved South African community with delays in early communication development.

The research question was ‘What is the relationship between certain environmental risks and communication delays in infants from an underserved community?’

## Research methods and design

A cross-sectional research design was used to explore the relationship between risks and communication delays in infants.

### Setting

Three clinics situated in underserved communities of the Tshwane district in the Gauteng province of South Africa (Olievenhoutbosch, Salvokop and Daspoort clinic) were utilised for data collection. Olievenhoutbosch clinic serves a population of 70 863 individuals residing in an area of 11.39 km^2^.^14^ Both Salvokop and Daspoort form part of the Pretoria subdistrict. The clinic situated in Salvokop area serves a population of 7123 and Daspoortclinic a population of 6355 individuals.^14^

### Participants

Convenience sampling was used as all of the parents or caregivers who visited the primary healthcare clinics for immunisation and health-related reasons over a three-month period were approached. The following inclusion and exclusion criteria were used: Only caregivers of infants aged between 6 and 12 months, who were proficient in Afrikaans or English, were asked to participate.

Two hundred and one infants were recruited (45% female), with similar age distributions for male (mean 8.68 months; standard deviation (SD) 1.86) and female infants (mean 8.73 months; SD 1.94). Ninety-four per cent of the participants resided in Olievenhoutbosch, whereas the remainder were from other areas such as Salvokop (2%), Daspoort (0.05%) and Mamelodi (0.5%). The majority of participants (98.5%) were black persons, with 1.5% of other ethnicities. Home language distribution in the study sample was as follows: Sepedi (33%), isiZulu (16%), Shona (11%), isiNdebele (10%), isiXhosa (6%), Southern Sotho (5%), Setswana (5%) and other (14%).

One-third (33%, *n* = 66) of mothers exited the educational system at Grade 10 or less, and 40% earned a monthly household income of less than R1500 ($150). One-third of the infants (33%, *n* = 66) had two or more siblings. Both parents were unemployed in 14% (*n* = 28) of cases and 77% (*n* = 154) lived in informal housing or stayed with others.

### Data collection tools and procedures

Data collection material included a structured interview schedule used to gain information from parents/caregivers, and a diagnostic communication assessment used to identify communication delays in infants. A structured interview schedule that consisted of closed-ended questions was developed to obtain participant background information, i.e. date of birth, duration of pregnancy, and gender, as well as the risk factors. Environmental risk factors that were investigated in the study were: level of education,^2,4,13,17^ housing status,^2,4,9^ age of mother at birth of infant,^3,4,9,10,17^ number of children,^1^^,^^4,17^ unemployment,^9,16,17^ average household income^9,16,17^ and gender of the infant.^4,5,20^

The Rossetti Infant Toddler Language Scale (RITLS) was used for the diagnostic communication assessment. The RITLS is a comprehensive, easy-to-administer and relevant tool to assess preverbal and verbal communicative abilities and interaction in infants and young children.^25^ Although this is a criterion-referenced tool, it has been widely used and validated in the past.^21,26,27,28,29,30^ The tool is designed to assess the following domains: pragmatics, gesture, play, language comprehension, language expression and interaction attachment.

The RITLS classifies infant development into three-month intervals, for instance 0–3 months and 3–6 months. At each interval developmental milestones under each of the domains are presented. When an infant at a specific age interval has one or more unmet milestone(s) in a specific developmental domain (such as language expression), the milestones of the previous interval are evaluated until the infant has met all the milestones at that age interval. The infant's developmental level is therefore the interval at which he or she obtained all the milestones within a developmental domain. It is therefore possible that the infant's developmental level is different for each of the evaluated domains; for example, an infant may present with a delay in expressive language and pragmatics, whilst the receptive language, interaction attachment and play skills are age-appropriate. An infant's progress is classified as delayed when one or more of the communication domains’ specific developmental levels differ by six months or more from the chronological age (for instance, when a 12-month-old infant's language expression scores on a 3–6-month developmental level, the infant presents with a communication delay).^25^

The first items in the ‘gesture’ subdomain only start at 9–12 months. Hence infants can only present with delays when they are 15 months or older. Since participants in the current study were all between six and 12 months of age and their development of gestures could not be classified as delayed, this subdomain was excluded from the results.

Prior to data collection ethical clearance was obtained from the Tshwane district research committee, Department of Health and the Faculty of Health Sciences and Humanities at the University of Pretoria. Parental and/or caregiver informed consent was obtained before data collection commenced. Both the interview and RITLS were carried out by the same speech-language pathologist, who has more than 10 years’ experience in the field. The structured interview with the parents/caregivers was conducted first. After the background information was obtained and the risks were identified by means of the interview schedule, the RITLS was completed by observing the infant during interaction with the parent and free play. If aspects of communication behaviour under investigation were not observed, the behaviour was elicited by the speech-language pathologist or the parent and/or caregiver's report on their infant's communicative behaviours was utilised to complete the RITLS.

Since the RITLS is a validated tool administration and scoring of the assessment was done by the same experienced speech-language pathologist to ensure reliability of data. Inter-rater reliability was also established, as independent raters observed 14% of the assessments and the outcomes of the tests were deemed similar to what the researcher obtained.

### Data analysis

To determine the existence of a significant association between risk factors and the outcome of the RITLS (indicating a communication delay or not) the Chi-square and Fisher's exact test statistics were used, with a significance level of *p* ≤ 0.05.

Only risk factors significantly associated with communication delays (*p* ≤ 0.05) were included in the second phase of the statistical analysis. Here a log-linear model analysis was used to model the probabilities of developing communication delays, taking into account both single and simultaneous effects of relevant risk factors. As the data (age of mother) were too limited to be added into the model in the categories < 18 years (*n* = 7), 19–34 years (*n* = 165) and < 35 years (*n* = 27), they were recategorised into two groups, namely 18–29 years and 35 years and older. Although a maternal age of 18–29 years is not an environmental risk, the effect of the age of the mother still needed to be explored.

For ease of interpretation the outcomes of the model were expressed as indices and converted into odds of communication delays for this specific combination of categories. Based on the odds the estimated probability of having a communication delay for a specific set of risk factors was calculated using the following formula:

$$\text{prob}=\frac{\text{odds}}{1+\text{odds}}$$ [Eqn 1]

## Results

A communication delay, as determined by the outcome of the RITLS, was present in 13% (*n* = 26/201) of the infants. The association of communication delay with each of the six risk factors constituted the first phase of the statistical analysis (Table 1). Three risk factors were found to be significantly associated with the prevalence of communication delays in the study population: (1) infants of mothers having three or more children showed a significantly higher prevalence of delays (sample percentage of 20%) than those of mothers having less than three children (10%) (Chi-square, *p* = 0.046); (2) having an informal housing status or staying with others was related to a marginally significantly lower prevalence in communication delays (10%) compared to when mothers have their own house (21%) (Chi-square, *p* = 0.052); and (3) the prevalence of communication delays in infants born of mothers aged 18 years or younger (43%) and 35 years or older (19%) was significantly higher than amongst those born of mothers between the ages of 19 and 34 years (11%) (Fisher's exact test, *p* = 0.04).

**TABLE 1:** Association of communication delay with psychosocial risk factors.

Risk factors	Delayed (%)	Significance (*p* value)	Test statistic
**Gender (*n* = 201)**			
Male (*n* = 111)	13	0.8797	Chi-square
Female (*n* = 90)	13		
Level of education (*n* = 200**)			
Grade 10 or less (*n* = 66)	18	0.1262	Chi-square
Grade 11–12, and/or tertiary (*n* = 134)	10		
**Number of children (*n* = 201)**			
2 or less (*n* = 135)	10	0.0458*	Chi-square
3 or more (*n* = 66)	20		
**Employment (*n* = 201)**			
Yes (*n* = 173)	12	0.2187	Fisher's exact
No (*n* = 28)	21		
**Housing status (*n* = 201)**			
Home owners (*n* = 47)	21	0.0516*	Chi-square
nformal housing or staying with others (*n* = 154)	10		
Average household income (*n* = 199**)			
Less than R1500 (*n* = 80)	11	0.6468	Chi-square
R1500 or more (*n* = 119)	13		
Age of mother at birth of youngest infant (*n* = 199**)			
18 yrs or less (*n* = 7)	43*	0.0397*	Fisher's exact
19–34 yrs (*n* = 165)	11*		
35 yrs and older (*n* = 27)	19*		

*, Statistically significant association (*p* ≤ 0.05); **, Numbers differ due to missing data

The outcome of the log-linear analysis in terms of indices and odds is shown in Table 2, with the three significant risk factors presented as combined factors. The indices were used to calculate the probabilities of both individual and combined risk factors by multiplying the overall main effect (index of the intercept) with one or more indices of the individual categories.

**TABLE 2:** Associated probability of single and combined risk factors predisposing to communication delay.

Parameter	Categories	Index	Odds	Probability *n* (%)
Housing status	Home owners	1.55	0.341	0.25 (25)
	Informal housing/staying with others	0.64	0.140	0.12 (12)
Age of mother and number of children	18–29 yrs, ≧ 3 children	1.90	0.418	0.30 (30)
	≧ 18 yrs, < 3 children	0.49	0.107	0.097 (10)
	≧ 30 yrs, ≧ 3 children	1.07	0.235	0.19 (19)
Age of mother and number of children and housing status	18–29 yrs, ≧ 3 children	1.90	0.647	0.39 (39)
Home owners	1.55			
	18–29 yrs, ≧ 3 children	1.90	0.267	0.21 (21)
	Informal housing/staying with others	0.64		
	≧ 18 yrs, < 3 children	0.49	0.167	0.14 (14)
	Home owners	1.55		
	≧ 18 yrs, < 3 children	0.49	0.068	0.06 (6)
	Informal housing/staying with others	0.64		
	≧ 30 yrs, ≧ 3 children Home owners	1.07	0.364	0.267 (27)
		1.55		
	≧ 30 yrs, ≧ 3 children	1.07	0.150	0.13 (13)
	Informal housing/staying with others	0.64		
Overall effect	**-**	0.22	-	-

There was a probability of 39% of having a communication delay for infants with two or more siblings, born of a mother aged 18–29 years who owns her own house. In contrast, when infants have none or only one sibling, and their caregivers own their house, irrespective of the age of the mother at birth, those infants had only a 14% risk of presenting with a communication delay (see Table 2). Table 2 summarises the associated probability for single and combined risk factors.

## Discussion

Prevalence of communication delay for infants aged 6–12 months (13%) in this study was high in comparison to the incidence of reported speech and language disability (5.6%) in children aged 0–2 years in the United Kingdom.^7^ The median prevalence of speech and language delays in children of two years of age reported in a systematic review was 5%.^8^ Variability in prevalence studies may be attributed to methodological differences and confounding factors such as risk exposure in study populations.^8,31^

The adverse impact of risks, specifically the number of siblings, on communication development in infants was demonstrated in the current study. This finding is in accordance with previous research that also confirmed that children with two or more siblings are at risk of communication delays.^12,32,33^ One of the possible reasons for younger siblings having delayed communication may be the fact that the older siblings are more verbal and may be speaking on behalf of the younger siblings.^12^ Also, larger families imply that parental interaction and attention is divided between the children, which may result in less attention and interaction than when there are only one or two children in the home. In 2006 the average fertility rate of black South African women was 2.9; as a result an average household will have approximately three children.^34^ In the current study one-third of the infants had two siblings or more, and it may be expected that these mothers will have another child in future, as 85% were 34 years of age or younger. This is in line with the fertility rate of 1.4 for 35–39-year-old black South African women.^34^ Developmental surveillance of infants who have two or more siblings may therefore be warranted in underserved communities.

Interestingly, infants living in homes owned by their parents had a higher probability (25%) of communication delay than those who lived in informal housing or with others (12%). Recent findings have demonstrated that the diversity of neighbourhoods in which infants live shapes their social learning independently of their caregiver and/or family interaction.^35^ The diverse neighbourhood of informal settlements or living in close proximity to others seemingly may aid social and communication development in infants. Consequently what was deemed a risk factor in the past^9^ may facilitate more opportunities for communication interactions and be conducive to social learning.

The impact of combined risk factors on communication development revealed that an infant was at greatest risk (39% probability) of developing a communication delay when: (1) mothers were between the ages of 18 and 29 years; (2) the parents own their own home; and (3) there are three or more children in the household. This information might allow primary healthcare workers, on the platform of community-oriented primary care,^36^ to identify infants at highest risk of communication delays in underserved communities in South Africa.

Considering that one in three infants were at risk of communication delay, the need for early communication intervention services, including developmental screening and comprehensive assessment and intervention, is evident. Completing a risk profile and conducting communication screening for infants could enable healthcare workers to identify at-risk infants and refer them for the required services. Such services may include creating awareness amongst parents on communication development and stimulation, and/or clinic and/or home-based early intervention. Internationally early intervention is becoming more prevention-orientated, encouraging individualising of children's learning experiences using evidence-based practices.^37^ Therefore implementing preventative strategies in at-risk populations in South Africa is well in line with the international focus of prevention-orientated early intervention services.

A study in the United States of America reported that 13% of infants were identified with developmental delays, but that only 10% of these infants received services by 24 months of age.^38^ Furthermore, black children were less likely to receive services than those from other ethnic and racial groups.^38^ It therefore appears that service delivery to at-risk infants is not only a local but also an international quandary, where disparities in service delivery to different ethnic and racial groups exist. Eradicating the gap in service delivery to improve availability of services to *all* infants at risk of communication delay should be advocated for in South Africa.

## Limitations

A limitation in the current study was that only caregivers or parents who were proficient in Afrikaans or English were included in the study. However, increased use of English in public administration, business and schools demonstrates the prominence of English in a variety of multilingual settings.^39^ Even though it is the first language of only 8.6% of South Africans, its wide demographic dispersal has resulted in English being the preferred medium for use within economic and social spheres.^39^ Still, since participants with limited or no verbal English or Afrikaans proficiency were excluded, the sample might not be entirely representative of the population sampled. It is therefore recommended that future research should be conducted on a randomised sample including all languages in underserved communities in South Africa.

## Conclusion

A clear relationship has been established in the current study between communication delay and three risk factors – age of the mother, number of children and housing status – in infants aged 6–12 months from these underserved communities. Furthermore, a combined effect of these risks accounted for a 39% probability of communication delay. As 13% of infants had a communication delay and more than one-third are at risk of developing communication delays in future, preventative strategies such as the implementation of a risk profile and a communication development screen should be implemented. This may ensure early identification of at-risk infants and assist healthcare workers in decision-making with regard to referral and preventative parental counselling.
